# Retraction: Selective inhibition of carbonic anhydrase IX over carbonic anhydrase XII in breast cancer cells using benzene sulfonamides: Disconnect between activity and growth inhibition

**DOI:** 10.1371/journal.pone.0326621

**Published:** 2025-06-18

**Authors:** The PLOS One Editors

After this article [1] was published, concerns were raised regarding results presented in Figs 2, 9, S4 and S7. Specifically:

- When levels are adjusted to visualize background, there appear to be regions around the bands in lanes 2–3 in the CA IX panel in Fig 2A that do not appear to match the overall background of the panel.
- The CA IX and CA II panels in Fig 2B appear similar to each other but the CA IX panel appears to have a region around the band in lane 2 that does not appear to match the overall background of the panel.
- Lanes 3 and 4 in the CA XII panel in Fig 2B appear similar to lanes 1 and 2 in the T47D CAXII panel in Fig 4A in [2].
- The following panels appear similar:
  - Fig 9A Migration NC in [1], Fig 9A Migration 10 μM U-CH_3_ in [1], Fig 6A Migration EV in [3] and Fig 6B UFH-001 in [4].
  - Fig 9A Invasion 100 μM U-CH_3_ in [1] and Fig 6A Invasion EV in [3].
  - Fig 9A Invasion 10 μM U-F in [1] and Fig 6A Invasion CA IX KD in [3].
  - Fig 9A Migration 100 μM U-NO_2_ in [1] and Fig 6A Migration CA IX KD in [3].
  - Fig 9A Invasion NC in [1] and Fig 6C UFH-001 in [4].
- S4 Figs A and B in [1] appear similar to the western blot inserts in Figs 4A and C in [3].
- The S7 Fig A CA XII, S7 Fig B CA IX and S7 Fig B CA II panels appear similar.
- There appears to be a vertical discontinuity between lanes 4 and 5 in the GAPDH panel in S7 Fig B.

Regarding the western blot concerns in S4 Fig, the corresponding author stated that the western blots in Figs 4A and C in [2] were intentionally reused in S4 Figs A and B in [1]. For the plate image concerns in Fig 9A, the corresponding author stated that the images in Fig 9A in [1] are correct, with the exception of the 10 μM U-CH_3_ panel which is incorrect and was duplicated from the control in Fig 9A in [1], and for which a replacement panel from the original experiments was provided. The corresponding author stated that the images in Fig 6A in [2] are incorrect. The author comments on the remainder of the concerns did not resolve the concerns. The corresponding author stated that with the exception of Figs 9B and C and the microarray data, the underlying data for this article [1] are no longer available.

In light of the nature and extent of the above concerns that question the integrity and reliability of the published results, and that cannot be resolved in the absence of the relevant underlying data, the *PLOS One* Editors retract this article.

FC and CTS did not agree with the retraction. SCF responded but expressed neither agreement nor disagreement with the editorial decision. MYM, ZC, AW, JVM, CT, KDB, MB, and RM either did not respond directly or could not be reached.

Owing to the concerns about similarities with previously published content [4], published 2018 Taylor & Francis which is not offered under a CC BY license, the Migration NC, Migration 10 μM U-CH_3_ and Invasion NC panels of Figure 9A are excluded from this article’s [1] license.
